# Effects of L-arginine on Nitric Oxide Synthesis and Larval Metamorphosis of *Mytilus coruscus*

**DOI:** 10.3390/genes14020450

**Published:** 2023-02-09

**Authors:** You-Ting Zhu, Lin-Li Liang, Tian-Tian Liu, Xiao Liang, Jin-Long Yang

**Affiliations:** 1 International Research Center for Marine Biosciences, Ministry of Science and Technology, Shanghai Ocean University, Shanghai 201306, China; 2 Key Laboratory of Exploration and Utilization of Aquatic Genetic Resources, Ministry of Education, Shanghai Ocean University, Shanghai 201306, China

**Keywords:** L-arginine, nitric oxide, *Mytilus coruscus*, larval metamorphosis, marine environmental factors, RNA interference

## Abstract

To investigate the regulatory functions of L-arginine and nitric oxide (NO) on *Mytilus coruscus* metamorphosis, *M. coruscus* larvae were exposed to an inhibitor of nitric oxide synthase (NOS), aminoguanidine hemisulfate (AGH), and a substrate for NO synthesis, L-arginine. We observed that NO levels showed a significant increase, and this trend continued with L-arginine treatment. When NOS activity was inhibited, the larvae could not synthesize NO, and metamorphosis was not inhibited even in the presence of L-arginine. On transfecting pediveliger larvae with *NOS* siRNA followed by L-arginine exposure, we found that the larvae did not produce NO and that the larval metamorphosis rate was significantly increased, suggesting that L-arginine regulates *M. coruscus* larval metamorphosis by promoting NO synthesis. Our findings improve our understanding of the effects of marine environmental factors on larval metamorphosis of mollusks.

## 1. Introduction

Marine invertebrates are prominent members of coastal and marine benthic communities. Their lifecycles are typically characterized by the planktonic larvae and benthic adult life stages [1,2,3]. In the early developmental stages, larvae swim freely in the ocean before settling and undergoing metamorphosis induced by environmental factors [2,4]. Larval settlement and metamorphosis are markedly affected by environmental conditions, as they influence their ability to successfully metamorphose, feed, and grow [5]. In addition, larval settlement and metamorphosis are affected by exogenous factors, such as physical, chemical, or biological parameters related to the substratum, particularly free amino acids from conspecific individuals, bacterial quorum-sensing molecules, and extracellular polymeric substances, such as polysaccharides, lipids, or proteins [4,5,6,7]. The metamorphosis of marine invertebrate larvae is also regulated by their neuroendocrine system, involving, for example, the adrenergic pathway, dopaminergic pathway, catecholamine biosynthesis, serotonergic pathway, cAMP analogs, cholinergic pathway, GABAnergic pathway, octopamine pathway, ecdysone pathway, amino acids, and various ions [8].

Nitric oxide (NO), synthesized by NO synthase (NOS), is a highly reactive and unstable free radical molecule [9]. As a pluripotent physiological messenger, NO plays various roles in transmitting nervous signals, in addition to participating in host immunity, regulating blood flow, and facilitating muscle relaxation in vertebrates [10]. In invertebrates, NO was first recognized to be involved in chemosensory and visual information processing and in the formation of long-term memory [11]. Since then, NO has been reported to play a role in the induction of cellular and humoral immune responses in insects [12,13,14]. Developmental biology studies have shown that NO plays a significant role in regulating larval settlement and metamorphosis in diverse marine invertebrates. In bryozoans [15], mollusks [16,17,18], annelids [19], arthropods [20,21], echinoderms [22], and tunicates [22,23], NO serves as an inhibitor of larval metamorphosis. However, in the sponge *Amphimedon queenslandica*, the solitary ascidian *Herdmania momus*, and the abalone *Haliotis asinine* [24,25,26], NO has been observed to induce larval metamorphosis. Furthermore, in the ascidian *Ciona intestinalis*, NO reportedly inhibits tail regression by inhibiting caspase-dependent apoptosis via the NO-cGMP signaling pathway [23]. Interestingly, it has been found that, in *Ciona* spp., activation of the MAP kinases ERK (Extracellular regulated protein kinases) and JNK (c-Jun N-terminal kinase) is pivotal for apoptosis and metamorphosis. Moreover, a decrease in NO levels downregulates the expression of MAP kinase phosphatases. It positively affects ERK signaling by reducing ERK phosphorylation [27,28,29,30]. These data indicate that NO regulates marine invertebrate metamorphosis via complex mechanisms, thereby making it worthy of investigating NO functions in different species.

NOS is a critical enzyme in the production of endogenous NO and catalyzes the conversion of L-arginine and NADPH to L-citrulline, NO, and NADP; consequently, NOS participates in regulating invertebrate metamorphosis [23,26,31,32]. In mammals, three isoforms of NOS are known, which are products of three distinct genes with specific expression profiles, cellular/subcellular localization, regulation, catalytic property, and inhibitor sensitivity [33,34]. In the past, these isoforms were differentiated on the basis of their constitutive (NOS I and III) versus inducible (NOS II) expression. At present, they are categorized as type I (NOS I, found in neuronal tissue), type II (NOS II, inducible in a wide variety of cells and tissues), and type III (NOS III, first found in vascular endothelial cells) [34,35,36]. Within metazoans, most invertebrate species have a single *NOS* gene [5,13,23,37,38,39,40,41,42,43,44]. In addition to NO synthesis, NOS mediates immune gene expression following lipopolysaccharide stimulation in *Bombyx mori* [13], nerve signaling in *Manduca sexta* [39], memory consolidation in *Lymnaea stagnalis* [45], tail regression in the metamorphosis process of *C. intestinalis* [23], immune defense against PAMPs and TNF-α in *Chlamys farreri* [46], and velum and foot development in *Crassostrea gigas* larvae [32]. Moreover, in *A. queenslandica*, symbionts supply their sponge larval host with the arginine required for NOS to synthesize NO [26]. NO synthetase is also involved in immune defense, neural signaling, and marine invertebrate metamorphosis and has complex biological functions. Considering this, comprehensively investigating the functions of NO synthetase seems essential.

The hard-shelled mussel (*M. coruscus*) inhabits the temperate zone of coastal waters in East Asia. It is an important commercial marine bivalve and fouling organism in the East China Sea, and its lifecycle includes the following stages: trochophore, D-shaped, umbo veliger, pediveliger, and post-larva [47]. We previously cloned the *NOS* cDNA sequence from *M. coruscus* (*McNOS*) and found mRNA levels to be downregulated in pediveliger larvae, which suggests that NO negatively regulates larval metamorphosis of *M. coruscus* [48]. However, direct evidence of NO production by NOS and its role in regulating larval metamorphosis remain to be obtained.

Herein, we investigated the ability of NOS to synthesize NO using exogenous L-arginine and its role in regulating larval metamorphosis of *M. coruscus*. We performed pharmacological and RNA interference (RNAi) experiments to generate evidence regarding the role of *NOS* in regulating metamorphosis of mussels.

## 2. Materials and Methods

### 2.1. Larval Culture and Spawning

Adult mussels (*M. coruscus*) were obtained from Gouqi Island, Zhejiang Province, China, and immediately transported to the laboratory on the same day. They were cleaned and cultured in a 10 L polycarbonate tank containing filtered (pore size: 0.45 μm) seawater (FSW) passed through a 1.2 μm acetate fiber filter, maintained at 21 °C, and fed a mixed diet of *Platymonas helgolandica* and *Isochrysis zhanjiangensis* every day.

Spawning was induced in the laboratory according to a previously described method, with slight modifications [49]. Briefly, mussels were placed in a sealed bag, which was incubated overnight on ice. Subsequently, they were transferred to a 2 L glass beaker containing FSW to release sperm and spawn. Fertilization was performed by gently mixing eggs and sperm suspensions in FSW and incubating for 20 min. Fertilized eggs were kept at 18 °C in FSW after removing excess sperm by washing with FSW on a nylon plankton net (20 µm mesh size). After 2 days, swimming larvae were fed *P. helgolandica* and *I. zhanjiangensis* at a density of 5 × 10^4^ cells/mL daily. Pediveliger larvae were collected, frozen in liquid nitrogen, and stored at −80 °C. These larvae were used to induce metamorphosis, as well as for NO content determination, RNAi, and gene quantification experiments. All animal handling procedures were approved by the Institutional Animal Care and Use Committee of Shanghai Ocean University.

### 2.2. Metamorphosis Assays of Pharmacological Treatments

Larvae were treated with epinephrine (EPI), which can induce larval metamorphosis; L-arginine, the substrate for NO synthesis; and aminoguanidine hemisulfate (AGH), which can inhibit NOS activity. Stock solutions of all chemicals were prepared as explained in the relevant manuals (EPI, 10^−3^ M; L-arginine, 10^−3^ M; and AGH, 10^−3^ M) using distilled water or 1 M HCl and diluted to desired final concentrations with autoclaved FSW (pH 7.8–8.2). All stock solutions and test chemical compounds were used immediately after preparation. The larval metamorphosis experiment was performed in a sterile glass petri dish (64.0 mm (Φ) × 19.0 mm) containing 20 pediveliger larvae (335.75 ± 16.5 μm (*n* = 100)) and 20 mL autoclaved filtered seawater (AFSW) mixed with chemicals. Larvae were treated with 10^−4^ M EPI, 10^−4^ M L-arginine, and 10^−3^ M AGH for 96 h. Larval behavior, the metamorphosis rate, and the survival rate were detected 24, 48, 72, and 96 h after chemical exposure. In case of mixed exposure experiments, 10^−3^ M AGH was added to the dish after treating the larvae with 10^−4^ mol/L L-arginine for 15 min. All bioassays were performed at 17 °C ± 1 °C in the dark with six replicates (*n* = 120). EPI was used as a positive control, and AFSW served as a negative control. The larvae were not fed any food during the experiments.

### 2.3. Determination of NO Levels in the Larvae

Each treatment group (*n* = 300 pediveliger larvae) was exposed to EPI, L-arginine, and AGH at the same concentrations as those stated in Section 2.2; the larvae were collected 72 h after chemical treatments. Four assessments were independently performed for each treatment group (*n* = 1200). The total protein was extracted from the larvae using a kit (CW0891M, Kangwei Century), and the protein concentration was determined with the modified BCA Protein Assay Kit (C503051, Shenggong, Shanghai, China). The NO levels in the larvae were measured using a kit (A012-1-2, Nanjing Jiancheng, Nanjin, China). All procedures were performed as recommended by the manufacturer.

For RNAi experiments, the siRNA-transfected pediveliger larvae were moved to the crystallization dish for temporary culture for 24 h. Then, the larvae were exposed to 10^−4^ M L-arginine for 48, 72, and 96 h, and the NO levels were determined. Each treatment group included 200 larvae, and three replicates were independently assessed (*n* = 600). All assays were performed in the dark at 17 °C ± 1 °C without algal feeding. AFSW was used as a negative control.

### 2.4. McNOS RNAi

The small interfering RNA (siRNA) sequence was designed and synthesized by GenePharma (Shanghai, China) based on *McNOS* full-length cDNA (Table 1). Larval RNAi was performed as previously described using the electroporation transfection method [50]. Pediveliger larvae (*n* = 200) were washed with AFSW and then transferred to 1.5-mL non-enzymatic centrifuge tubes containing 1 mL AFSW (Control, negative control group), 1 mL AFSW and 1.2 μg non-target gene siRNA (NC siRNA, negative control group), and 1 mL AFSW and 1.2 μg target gene siRNA (*McNOS* siRNA, experimental group), followed by incubation for 5 min. Subsequently, the pediveliger larvae and siRNA mixture were transferred to a 0.4 cm diameter cuvette (Bio-Rad, Hercules, CA, USA), and electroporation was performed using a Gene Pulser Xcell electroporator (Bio-Rad). After incubation at room temperature for 10 min, the larvae were transferred to AFSW for culture. Each treatment group included 200 larvae, and three replicates were independently assessed (*n* = 600). In total, 120 larvae from each treatment group were examined for the survival rate after transfection with siRNA at 24, 48, 72, and 96 h. Pediveliger larvae after RNAi was used for metamorphosis induction, gene quantification, and NO content determination.

### 2.5. Total RNA Extraction and Quantitative Real-Time PCR

The total RNA from larvae was extracted using TRIzol (Invitrogen, Carlsbad, CA, USA), according to manufacturer instructions, and quantified using NanoDrop One (Thermo Scientific, Waltham, MA, USA). The RNA quality was assessed using 1% agarose gel electrophoresis. For gene expression profiling analysis, 1000 ng total RNA was reverse-transcribed using the Transcriptor First Strand cDNA Synthesis Kit (Roche, Basel, Switzerland), as per manufacturer instructions.

qRT-PCR was performed to analyze *McNOS* mRNA expression levels. The gene encoding elongation factor-1a (*EF*-*1α*) served as a reference for normalization. Fluorescence quantitative primers of *NOS* were based on *McNOS* full-length cDNA (GenBank ID: MN153299) and were designed and synthesized by Shanghai Shenggong (Table 1). Gene expression analysis involved four independent experimental groups, with each containing 200 pediveliger larvae (*n* = 800). Three technical replicates were assessed for each sample. qRT-PCR was performed in a 96-well plate on LightCycler 960 (Roche, Basel, Switzerland).

A standard curve was generated for each qRT-PCR over a range of 10^7^–10^1^ copies of the target amplicon. The Ct value of each sample was used in conjunction with the standard curve to determine the copy number of the amplified target genes. Each qRT-PCR mixture (10 mL) contained 5 mL 2 × FastStart Essential DNA Green Master (Roche), 0.3 mL of forward and reverse primers each (final concentration, 300 nM), 3.4 mL deionized water, and 1 mL template cDNA. The cycling conditions were as follows: initial denaturation at 95 °C for 10 min, followed by 45 cycles at 95 °C for 10 s, 60 °C for 10 s, and 72 °C for 10 s. To verify specificity, melting curve analysis was performed with a temperature gradient of 0.5 °C/s from 65 °C to 95 °C. qRT-PCR efficiency varied between 90% and 105%, with R^2^ ≥ 0.99. The relative abundance of target gene in different samples was assessed using JMP 10.0, and differences were evaluated by using one-way analysis of variance. *p* < 0.05 indicated a significant difference.

### 2.6. Metamorphosis Assays and Survival Rate of Pediveliger Larvae after McNOS RNAi

The siRNA-transfected pediveliger larvae were moved to the crystallization dish for temporary culture for 24 h. Then, 20 larvae were randomly selected and placed into a glass Petri dish with 20 mL AFSW containing 10^−4^ M L-arginine. The larval metamorphosis rate was determined at 48, 72, and 96 h after treatment using a previously reported method [51]. Pediveliger larvae treated with AFSW were used as a negative control, and 10^−4^ M EPI served as a positive control. Each treatment group included 20 larvae, and six replicates were independently assessed (*n* = 120). All assays were performed in the dark at 17 °C ± 1 °C without algal feeding.

### 2.7. Statistical Analysis

Data were analyzed using JMP 10.0 (SAS Institute, Cary, NC, USA). The percentage of metamorphosed larvae was first arcsine-transformed, and normality and homogeneity were then tested. The Wilcoxon/Kruskal–Wallis tests were used to analyze data obtained from larval metamorphosis assays and qRT-PCR. *p* < 0.05 indicated a significant difference.

## 3. Results

### 3.1. NO Content in the Larvae on Exposure to L-arginine and AGH

**Figure 1:**
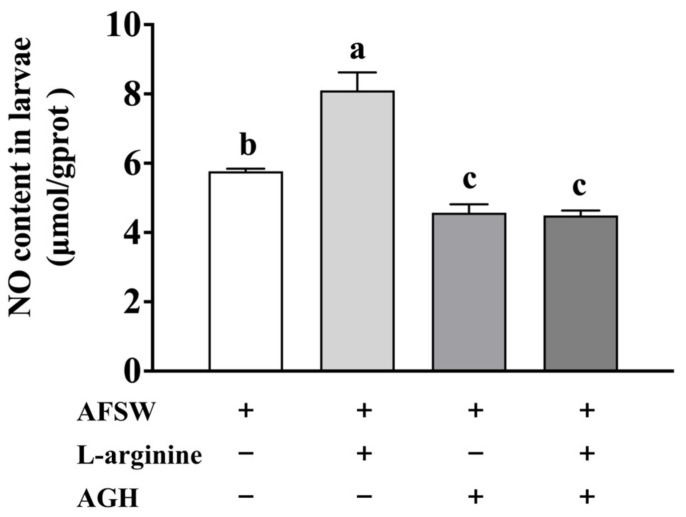
NO level in larvae after L-arginine and AGH exposure for 72 h. Values represent mean ± SEM (*n* = 120), and bars with different letters indicate significant differences (*p* < 0.05).

To verify the function of NOS in *M. coruscus*, pediveliger larvae were treated with L-arginine and AGH. In comparison to the control (AFSW), NO levels showed a significant increase after L-arginine exposure for 48 h, whereas the inhibition of NOS activity by AGH led to a decrease in NO levels irrespective of subsequent L-arginine treatment (Figure 1). It is notable that NOS activity inhibition by AGH followed by L-arginine exposure did not increase NO levels (Figure 1). These results suggested that NOS can synthesize NO using the exogenous substrate L-arginine.

### 3.2. Effects of L-arginine on NOS Expression

**Figure 2:**
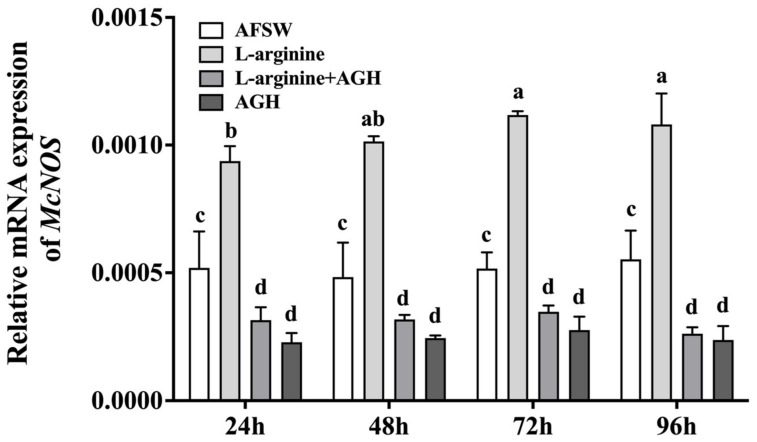
*NOS* mRNA expression levels at different timepoints after L-arginine and AGH exposure. Values represent mean ± SEM (*n* = 120), and bars with different letters indicate significant differences (*p* < 0.05).

To investigate the effects of L-arginine on *NOS* expression, the larvae were exposed to L-arginine and AGH, and *McNOS* mRNA expression was measured using qRT-PCR. After 24–96 h of L-arginine exposure, *McNOS* expression in the larvae was found to be significantly upregulated as compared to that in the control (AFSW), with no significant difference between different treatment times. When the larvae were exposed to L-arginine for 24–96 h after AGH treatment, *McNOS* expression was similar to that in the AGH exposure group and significantly downregulated as compared to that in the L-arginine exposure and control (AFSW) groups (Figure 2).

### 3.3. Behavior of the Larvae on the Exposure to L-arginine and AGH

**Figure 3:**
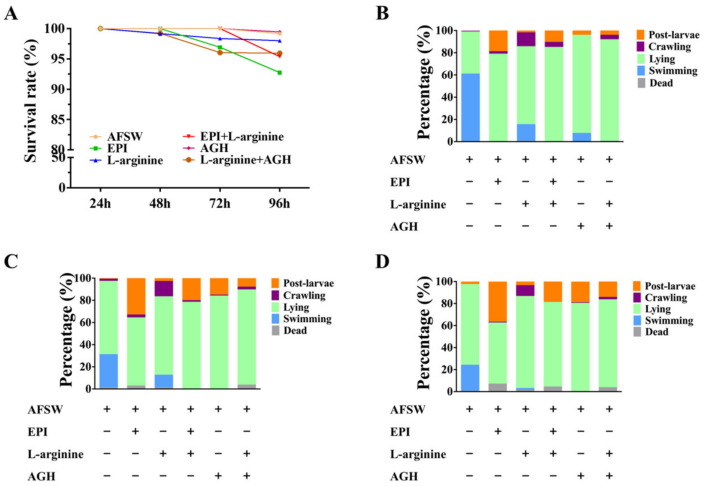
Survival rate and behavior of the larvae exposed to L-arginine and AGH. (**A**) Survival rates after exposure to L-arginine and AGH for 24, 48, 72, and 96 h. (**B**) Larval behavior on exposure to L-arginine and AGH for 48 h, (**C**) 72, and (**D**) 96 h.

To determine the effects of NO and NOS on larval behavior and metamorphosis, the larvae were stimulated with EPI (positive control), L-arginine, and AGH. Neither of them had a significant effect on the survival rate of the larvae (Figure 3A). With an increase in exposure time, as compared to the control (AFSW), larval metamorphosis showed an increase; the least increase was observed on L-arginine exposure. The proportion of the larvae in metamorphosis induced by EPI was reduced in the presence of L-arginine. When the larvae were exposed to L-arginine alone, more larvae remained in the crawling stage, representing the beginning of metamorphosis preparation. Meanwhile, the proportion of metamorphotic larvae showed an increase from 48 to 72 h after AGH exposure, while L-arginine reduced larval metamorphosis in the presence of AGH (Figure 3B–D).

### 3.4. Metamorphosis of Pediveliger Larvae on Exposure to L-arginine and AGH

**Figure 4:**
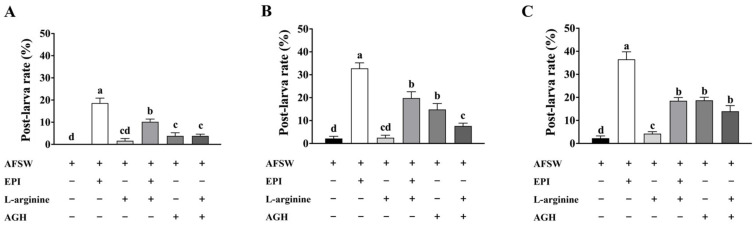
The larval metamorphosis rate on exposure to L-arginine and AGH. (**A**) Larvae exposed to L-arginine and AGH for 48 h, (**B**) 72 h, and (**C**) 96 h. Values represent mean ± SEM (*n* = 120), and bars with different letters indicate significant differences (*p* < 0.05).

The larval metamorphosis rate showed a significant increase after 48 h of EPI (up to 18.63%) and AGH (up to 3.82%) exposure compared to the control (AFSW). The addition of L-arginine after the induction with EPI significantly inhibited the larval metamorphosis rate, with the metamorphosis rate decreasing to 10.17% (Figure 4A). After 72 h, the larval metamorphosis rate reached 32.78% under EPI and 14.85% under AGH induction. However, the induction rate of EPI and AGH was only 19.81% and 7.65%, respectively, in the presence of L-arginine (Figure 4B). After 96 h of induction, the larval metamorphosis rate reached 36.52% under EPI and 18.73% under AGH induction. In the presence of L-arginine, the induction rate of EPI was still only 18.73%, but that of AGH increased to 13.95% (Figure 4C).

### 3.5. NOS siRNA and Survival Rate of the Larvae

**Figure 5:**
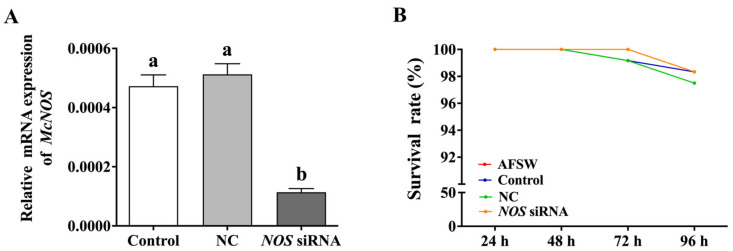
*NOS* mRNA expression and the larval survival rate after the knockdown of *NOS* expression. (**A**) *McNOS* mRNA expression in the larvae after *NOS* siRNA transfection for 48 h. (**B**) Larval survival rates at different timepoints after *NOS* siRNA transfection. Values represent mean ± SEM (*n* = 800), and bars with different letters indicate significant differences (*p* < 0.05). Control: the larvae were untreated, NC: the larvae were transfected with 1.2 μg non-target gene siRNA, *NOS* siRNA: the larvae were transfected with 1.2 μg *McNOS* siRNA.

In our previous study, we cloned *NOS* from *M. coruscus* (i.e., *McNOS*); however, we did not comprehensively investigate its function [48]. To determine the role of *McNOS* in the synthesis of NO as well as the regulation of larval metamorphosis, RNAi was performed to knockdown *McNOS* expression. We found that NOS siRNA treatment significantly suppressed *McNOS* expression until 48 h post-treatment, and the expression of *McNOS* decreased 75.66% compared to that in the control group (Control) (Figure 5A). Meanwhile, siRNA transfection did not cause larval death; the survival rate of each treatment group was at least 97.5% (Figure 5B).

### 3.6. NO Content in the Larvae after McNOS siRNA and L-arginine Treatment

**Figure 6:**
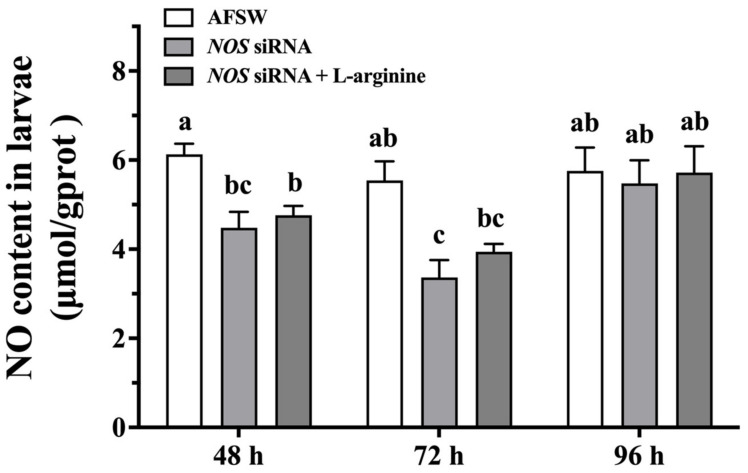
NO content in the larvae at different timepoints after *NOS* siRNA transfection. Values represent mean ± SEM (*n* = 800), and bars with different letters indicate significant differences (*p* < 0.05).

To determine the role of NOS and L-arginine in NO production, pediveliger larvae were treated with RNAi and L-arginine, and then NO levels were measured. The NO levels at 48 h and 72 h after *McNOS* RNAi (*NOS* siRNA) were approximately 4.48 μmol/gprot and 3.37 μmol/gprot, respectively, showing a significant decrease as compared to that in the control (AFSW); the NO levels returned to normal levels at 96 h (Figure 6). However, exposure of the larvae to L-arginine after siRNA transfection did not increase NO levels (Figure 6), indicating that that when NO synthetase function is absent, the larvae are unable to synthesize NO even in the presence of L-arginine.

### 3.7. The Larval Metamorphosis Rate after McNOS siRNA and L-arginine Treatment

**Figure 7:**
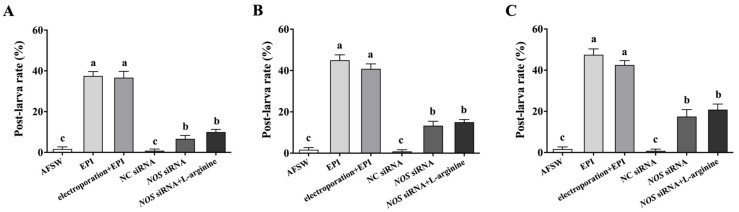
The larval metamorphosis rate after treatment with L-arginine and *NOS* siRNA transfection. (**A**) The larval metamorphosis rate on exposure to L-arginine for 48 h, (**B**) 72 h, and (**C**) 96 h. Values represent mean ± SEM (*n* = 120), and bars with different letters indicate significant differences (*p* < 0.05).

To conclusively determine the role of NOS and L-arginine in larval metamorphosis, we investigated the larval metamorphosis rate after RNAi and L-arginine treatment. The larval metamorphosis rate was 37.5%, 45%, and 47.5% after 48, 72, and 96 h of EPI exposure, respectively, which was significantly higher than those in the control (AFSW). Furthermore, the larval metamorphosis rate in the EPI exposure with only electroporation group (electroporation without siRNA) was 36.67%, 40.83%, and 42.5% at 48, 72, and 96 h post-treatment, respectively, which showed no significant differences in comparison to the rates in the EPI exposure group. In contrast, after *NOS* siRNA transfection, although the larval metamorphosis rate was significantly higher than that in the control (6.67%, 12.5%, and 17.5% at 48, 72, and 96 h after transfection, respectively), the rate significantly decreased relative to that in the EPI exposure group. When larvae were exposed to L-arginine after *NOS* interference, the larval metamorphosis rate was 10%, 14.17%, and 20.83%, respectively, after 48, 72, and 96 h of siRNA transfection, showing no significant differences compared with the rates in the only *NOS* RNAi group (Figure 7).

## 4. Discussion

Metamorphosis is a widespread life history strategy of marine invertebrates, which often involves dramatic morphological, physiological, behavioral, and ecological transformations between the larvae and the juvenile [52,53]. Previous studies have elucidated the effects of various environmental factors, including amino acids such as L-arginine, on larval metamorphosis of marine invertebrates. Herein, we provide evidence that L-arginine serves as a critical endogenous regulator of *M. coruscus* larval metamorphosis by controlling the function of NOS, which reportedly inhibits larval metamorphosis [48].

Arginine is synthesized from citrulline in the urea cycle by the sequential action of the cytosolic enzymes argininosuccinate synthetase and argininosuccinate lyase [54]. It is involved in diverse metabolic pathways and plays a key role in physiological and pathological processes. It produces a wide range of metabolites in mammals, including being catalyzed by NOSs as a precursor, and produces nitric oxide [55,56]. In most invertebrates, arginine cannot be biosynthesized but is instead acquired from exogenous sources [57], such as from biofilm-forming [58,59] or symbiotic [26] bacteria. In this study, *M. coruscus* larvae were treated with L-arginine to investigate whether it is utilized as a substrate for NO synthesis. We found that NO levels in larvae significantly increased in the presence of L-arginine, whereas NO levels did not increase when NOS activity was inhibited by an NOS inhibitor, indicative of the utilization of exogenous L-arginine by *M. coruscus* larvae for NO production. These findings suggest that utilizing L-arginine as a substrate for NO synthesis is an essential function in many organisms, including *M. coruscus*.

Most previous studies using exogenous L-arginine as a substrate to synthesize NO have focused on investigating the regulatory functions of NO on metamorphosis. However, L-arginine and NO have opposite effects on regulating metamorphosis in different species. For instance, studies have revealed that exogenous L-arginine can induce metamorphosis of the ascidian *H. momus* [24] and the abalone *Haliotis asinina* [25]. However, it has been found to inhibit metamorphosis in the nudibranch *Phestilla sibogae* [18] and the sea slug *Alderia willowi* [60]. Furthermore, in the sponge *A. queenslandica*, bacterial symbionts biosynthesize arginine, which is used by NOS to produce NO; this NO then positively regulates larval metamorphosis [26]. We observed that *M. coruscus* larval metamorphosis was significantly inhibited when NO levels increased upon the addition of L-arginine. These inhibitory effects of L-arginine could be alleviated by inhibiting NOS activity in advance, which suggests that L-arginine is able to produce NO via NOS, consequently inhibiting larval metamorphosis.

In marine invertebrates, metamorphosis can be usually divided into two core processes: settlement and metamorphosis. A study based on the tube-dwelling polychaete worm *Hydroides elegans* reported that the larvae actively swim near the surface of a suitable substrate, settle on it, and begin to metamorphose [5]. Different environmental factors and endogenous factors regulate larval settlement and metamorphosis, respectively. For example, EPI can induce *Hydroides ezoensis* larval metamorphosis but not settlement [61]. Similarly, norepinephrine can induce *C. gigas* larval metamorphosis but not settlement [62]. In *C. gigas*, NO evidently detains larvae in the larval stage by promoting a swimming and crawling behavior instead of metamorphosis [32]. An earlier study showed that *H. elegans* settlement is induced by a selection of bacterial species present in a natural biofilm [5]. Another study reported that two cues from a bacterial biofilm, one waterborne and one surface-bound, synergistically act in inducing the larval metamorphosis of the mussel *Mytilus galloprovincialis* [63]. Our data indicate that on L-arginine exposure, a higher number of *M. coruscus* larvae settle and crawl on the bottom of the Petri dish, but not metamorphose, suggesting that L-arginine mainly inhibits larval metamorphosis rather than affecting crawling behavior. This is similar to the positive effect of EPI on *H. ezoensis* larval metamorphosis but contradictory to the observations in Pacific oysters [32,61].

NOS is a key rate-limiting enzyme in the synthesis of NO in vivo [34]. *NOS* genes have been cloned and identified from >46 species, including humans [64]. In mollusks, NOS function has been explored in the context of metamorphosis regulation, immune defense, and nerve signal transmission. In particular, the ability of NOS to promote or inhibit larval metamorphosis has been extensively studied, which is related to its function of NOS. For instance, *NOS* was characterized in the pond snail *L. stagnalis* and prosobranch *Stramonita haemastoma* and found to be involved in modulating the central neural network underlying feeding and the nervous system [65,66]. Furthermore, molecular cloning of NOS mRNA from the cuttlefish *Sepia officinalis* was reported. In situ hybridization showed its expression in the immature and mature cells of the ink gland, as well as in the regions of the nervous system associated with the ink defense system [42]. NOS has been identified in the scallop *C. farreri* and sea cucumber *Apostichopus japonicus*, and its immune defense functions against pathogen-associated molecular patterns and bacteria were investigated [46,67]. *NOS* expression in *C. gigas* was recently reported primarily in the velum and foot, which are key larval organs, and *NOS* was speculated to be involved in regulating the swimming or crawling behavior and neuroendocrine downstream responses [32]. In our previous study, we cloned *NOS* from *M. coruscus* and analyzed its expression in the different developmental stages of larvae and tissues of adult mussels. However, no conclusive evidence was obtained to prove its function in the larvae [48]. Herein, we knocked down the expression of *NOS* using RNAi and found that NO levels in the larvae significantly decreased even when L-arginine was added. Moreover, the larval metamorphosis rate significantly increased, and L-arginine could not inhibit larval metamorphosis. On L-arginine exposure, the NOS expression was significantly upregulated. Altogether, these data suggest that L-arginine induces NOS to synthesize NO, which then inhibits *M. coruscus* larval metamorphosis.

As mentioned in the introduction, NO could inhibit tail regression by inhibiting caspase-dependent apoptosis via the NO-cGMP signaling pathway in the *C. intestinalis* [23]. It is suggested that the inhibition of L-arginine and NO on larval metamorphosis of *M. coruscus* is likely achieved by inhibiting apoptosis mediated by the cGMP-caspase-3 pathway. On the other hand, neurotransmitters such as EPI, L-3,4-dihydroxyphenylalanine (L-DOPA), and serotonin can influence larval metamorphosis of bivalve mollusks by regulating intracellular cAMP concentrations through their binding to transmembrane G-protein-coupled receptors (GPCRs) (adrenergic, dopaminergic, and serotonergic receptors) [8]. Our previous studies demonstrated that G protein-coupled receptors and signal transduction via the AC/cAMP pathway could mediate larval metamorphosis of *M. coruscus* [68]. Furthermore, EPI was found to induce larval metamorphosis through α2-adrenergic receptor [50]. Interestingly, cGMP can regulate cAMP concentration and cAMP/PKA signaling in human hearts by activating Phosphodiesterase 2 (PDE2) or inhibiting Phosphodiesterase 3 (PDE3) [69]. Therefore, understanding the molecular mechanism of NO and neurotransmitters regulating larval metamorphosis, as well as the interaction between their downstream cGMP and cAMP, is crucial to elucidate the regulation of bivalve metamorphosis.

## 5. Conclusions

In our study, we elucidated the crucial role of L-arginine as an endogenous and negative regulator of metamorphosis of *M. coruscus*. L-arginine was found to induce the expression of *NOS* in *M. coruscus* larvae and was utilized by NOS to synthesize endogenous NO. Although NO did not affect the larval crawling behavior, it inhibited larval metamorphosis. L-arginine addition increased NO levels in the larvae, with a large number of larvae remaining in the crawling stage without metamorphosis. In addition, we confirmed the function of NOS in the larvae by using RNAi. When NOS function was inhibited, the larvae were unable to synthesize NO even in the presence of L-arginine, and larval metamorphosis was accordingly not inhibited. Our data provide valuable new information regarding larval metamorphosis and emphasize the importance of the L-arginine–NOS–NO pathways during this key developmental stage of bivalves.

## Figures and Tables

**Table 1:** Primer sequences used for *M. coruscus NOS* mRNA expression analysis.

Primer	Sequence (5′–3′)	Usage
NOS siRNA	CGGCCGUUGACUAUAUAUATT	RNAi
NC siRNA	UUCUCCGAACGUGUCACGUTT	RNAi
*McNOS*-RT-F	GAAGTTCAAATAAAGCATCCAAAAT	qRT-PCR
*McNOS*-RT-R	GTGGTGGTCCATTATTGTAGCTCCT	qRT-PCR
*EF*-*1α*-RT-F	CACCACGAGTCTCTCCCTGA	qRT-PCR
*EF*-*1α*-RT-R	GCTGTCACCACAGACCATTCC	qRT-PCR

## Data Availability

The original contributions presented in the study are included in the article.
